# *XGANDALF* – extended gradient descent algorithm for lattice finding

**DOI:** 10.1107/S2053273319010593

**Published:** 2019-08-30

**Authors:** Yaroslav Gevorkov, Oleksandr Yefanov, Anton Barty, Thomas A. White, Valerio Mariani, Wolfgang Brehm, Aleksandra Tolstikova, Rolf-Rainer Grigat, Henry N. Chapman

**Affiliations:** a Center for Free-Electron Laser Science, Deutsches Elektronen-Synchrotron DESY, Notkestraße 85, 22607 Hamburg, Germany; b Institute of Vision Systems, Hamburg University of Technology, Harburger Schloßstraße 20, 21079 Hamburg, Germany; c Department of Physics, Universität Hamburg, Luruper Chaussee 149, 22761 Hamburg, Germany; d The Hamburg Center for Ultrafast Imaging, Universität Hamburg, Luruper Chaussee 149, 22761 Hamburg, Germany

**Keywords:** indexing, *XGANDALF*, *CrystFEL*, multiple lattices, serial crystallography

## Abstract

Serial crystallography records still diffraction patterns from single, randomly oriented crystals, then merges data from hundreds or thousands of them to form a complete data set. To process the data, the diffraction patterns must first be indexed, equivalent to determining the orientation of each crystal. A novel automatic indexing algorithm is presented, which in tests usually gives significantly higher indexing rates than alternative programs currently available for this task. The algorithm does not require prior knowledge of the lattice parameters but can make use of that information if provided, and also allows indexing of diffraction patterns generated by several crystals in the beam. Cases with a small number of Bragg spots per pattern appear to particularly benefit from the new approach. The algorithm has been implemented and optimized for fast execution, making it suitable for real-time feedback during serial crystallography experiments. It is implemented in an open-source C++ library and distributed under the LGPLv3 licence. An interface to it has been added to the *CrystFEL* software suite.

## Abstract 2

A description and evaluation are given of *XGANDALF*, extended gradient descent algorithm for lattice finding, an algorithm developed for fast and accurate indexing of snapshot diffraction patterns.

## 1. Introduction   

Serial crystallography (SX) experiments (Chapman *et al.*, 2011 ▸; Schlichting, 2015 ▸) record a sequence of diffraction patterns, each from a different crystal in a random and unknown orientation. Measurements from hundreds or many thousands of crystals are used to build up a complete data set. Experiments usually aim to measure not more than one crystal per diffraction pattern, although the contribution of multiple crystals in a single diffraction measurement is not uncommon. The difference in the measurement approach compared with conventional rotation crystallography has necessitated the development of new software for processing SX data, with several software packages now available including *CrystFEL* (White *et al.*, 2012 ▸), *DIALS* (Winter *et al.*, 2018 ▸) and *nXDS* (Kabsch, 2014 ▸). The main steps in analysis involve Bragg spot detection, indexing diffraction patterns, integration of detector counts in Bragg reflections, and merging of data from all individual crystals into a common data set. A key step is indexing the Bragg spots observed in a pattern, which is required to integrate and scale Bragg intensities into a common lattice and to predict the locations of other Bragg spots to be included in this merging process. Several automatic indexing algorithms have been developed and implemented in widely used software like *MOSFLM* (Powell, 1999 ▸), *XDS* (Kabsch, 1993 ▸, 2010 ▸), *DirAx* (Duisenberg, 1992 ▸) and *LABELIT* (Sauter & Zwart, 2009 ▸). Although originally devised for rotation-series data, these algorithms are also capable of indexing snapshot diffraction patterns. Other algorithms have been devised specifically for snapshot data (Ginn *et al.*, 2016 ▸; Gildea *et al.*, 2014 ▸).

In our SX experiments we often observe crystal diffraction patterns which appear to correspond to crystal lattices but nonetheless cannot be indexed by the existing approaches. Even when several different indexing algorithms are applied to each pattern, only a fraction of the frames can be indexed. Patterns with small numbers of Bragg spots, large amounts of background noise that lead to spurious peaks in the Bragg peak detection stage, or with multiple overlapping crystal diffraction patterns (‘multiple hits’) are particularly problematic and often cannot be indexed by current algorithms. In principle, it should be possible to index every diffraction pattern provided that Bragg spot locations are consistent with a true crystal diffraction pattern rather than spurious noise. It thus appears advantageous to deviate from previous approaches adapted from indexing rotation data and instead develop an algorithm for the express purpose of indexing SX crystal diffraction patterns. We set out to develop such a new and computationally efficient algorithm for indexing SX crystal diffraction patterns with the aim of maximizing the indexing rate while being robust to outliers.

Indexing involves identifying the diffraction order of all Bragg spots measured in a diffraction pattern, equivalent to determining the crystal orientation. In most indexing algorithms, the process begins by mapping the positions of Bragg spots found on the detector to radiation scattering momentum transfer vectors 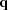 in the three-dimensional (3D) reciprocal space using prior information about the detector geometry (including sample-to-detector distance) and the wavelength of the incident beam. The resulting points in 3D reciprocal space approximate the points of the reciprocal lattice, which is initially unknown. We call these points ‘nodes’ to abstract the problem from crystallographic indexing to the more general problem of fitting a lattice to noisy locations. One possible approach to indexing is to detect maxima in the Fourier transform of the pattern of nodes (Steller *et al.*, 1997 ▸). Such maxima mark the directions with maximum periodic repetition, which can form the basis vectors of the wanted lattice. This approach is taken in *DIALS* (Gildea *et al.*, 2014 ▸) and *MOSFLM* (Powell, 1999 ▸). The related *DirAx* algorithm finds principal repeat directions by searching for frequently occurring repeats perpendicular to triplets of nodes (Duisenberg, 1992 ▸). Another popular indexing approach is to search for frequently occurring difference vectors between the nodes, as is done in *XDS* (Kabsch, 2010 ▸) and *TakeTwo* (Ginn *et al.*, 2016 ▸). The *FELIX* indexer (Beyerlein *et al.*, 2017 ▸) uses a different approach which is to map the set of possible crystal orientations that are consistent with particular Bragg spots to lines in Rodrigues–Frank space to find a consensus orientation for all peaks.

**Figure 1:**
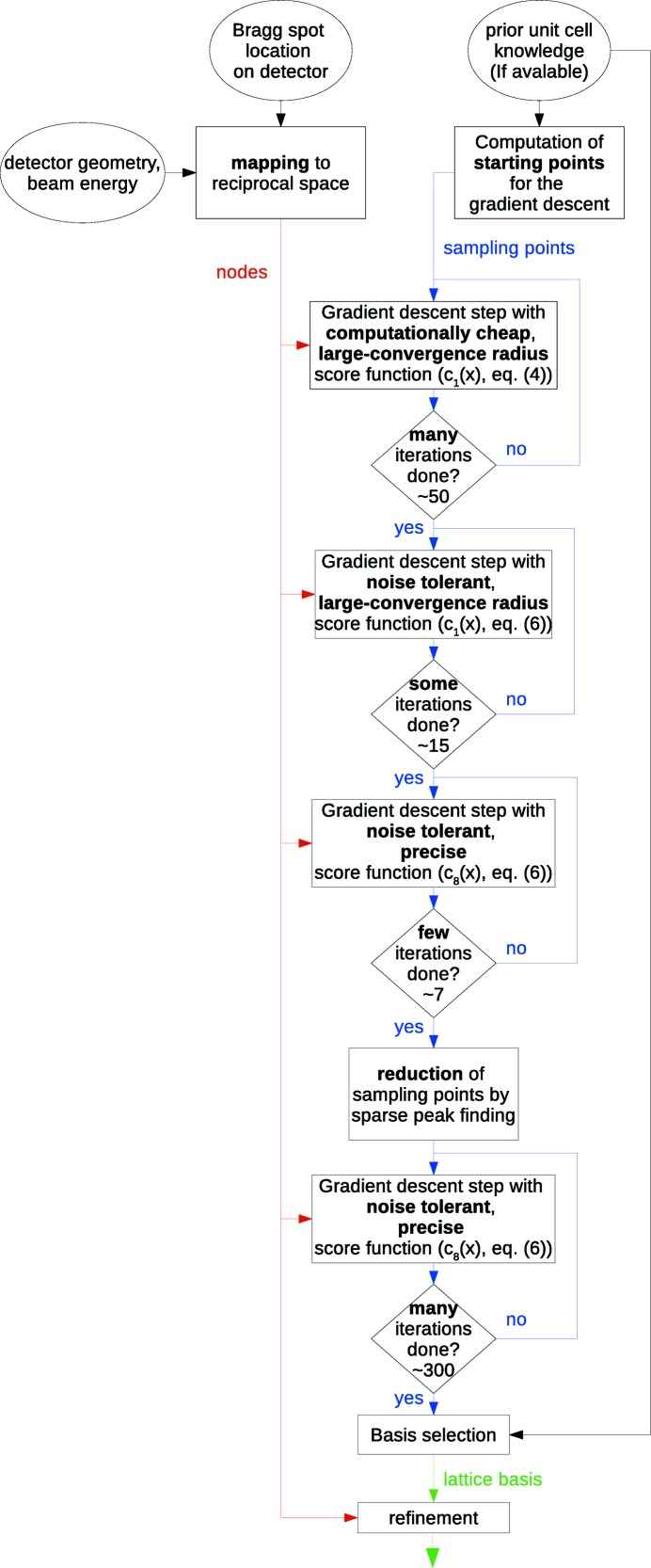
Overall structure of the *XGANDALF* algorithm.

Our algorithm can be considered as a modified version of the Fourier methods. To improve noise tolerance, we replace the Fourier transform by a similar transform that uses periodic basis functions combined with a non-linear weighting scheme. To achieve fast execution, the algorithm employs a multi-step heuristic, *i.e.* an approximate but efficient method, based on an extended gradient approach to identify maxima in the transformed pattern of nodes corresponding to points on the real-space crystal lattice. The real-space lattice basis is then formed from these maxima, while maximizing the number of the observed nodes that are consistent with that basis choice and minimizing the distances between those nodes and their closest lattice point. An overview of the main steps of the proposed algorithm is provided in Fig. 1 ▸.

We call our algorithm *XGANDALF*, eXtended GrAdieNt Descent Algorithm for Lattice Finding.

## 2. Algorithm description   

### 2.1. Overview   

**Figure 2:**
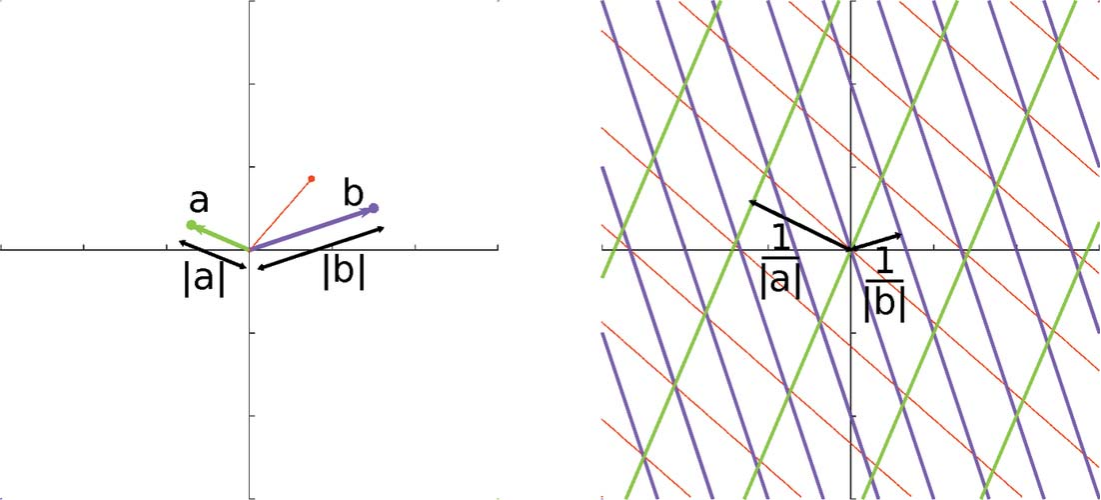
Line series in real space (green and purple, right panel) generated by two nodes *a* and *b* in the 2D reciprocal space (left). The distances between adjacent parallel lines are given by the familiar reciprocal of the distance of magnitude of the momentum transfer of the node. A third node (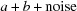, red) is shown, along with a corresponding set of lines in the right panel, to show that in the presence of noise there usually are no points where all sets of lines intersect.

The indexing algorithm determines the Miller indices of a number of observed Bragg peaks in a snapshot diffraction pattern, given knowledge of the experiment geometry and optionally the unit-cell parameters of the crystal. It consists of the following key steps:

(i) Bragg spot locations on the detector are mapped to positions in reciprocal space. We call the location of the momentum transfer vector of a Bragg spot a node, to distinguish it from the exact reciprocal-lattice points, the locations of which are initially unknown.

(ii) Each node in reciprocal space is used to define a set of equidistant parallel planes in 3D real space, as shown in Fig. 2 ▸. Intersections of parallel planes generated from different nodes are solutions to the indexing problem. The rest of the algorithm is devoted to finding these intersections in the presence of noise, spurious peaks and multiple lattices.

(iii) Continuous ‘proximity functions’ based on distance to each node’s parallel planes are defined and summed to create a score function to find the points of intersection. Intersections of planes become maxima of the score function, with the continuous score function serving to suppress the effect of experiment noise and inaccuracies. A series of progressively sharper and steeper proximity functions are used with the result that spurious nodes corresponding to falsely identified Bragg peaks or reflections belonging to competing lattices are removed from the set of Bragg reflections that are used to generate the nodes.

(iv) A heuristic (a fast technique for finding approximate solutions) is used to find maxima of the score function. Sharper proximity functions require more computations to find maxima, hence we choose more computationally efficient proximity functions to reduce the search space early in the computation. An extended gradient descent method is applied to migrate the starting points to the maxima of the score function and avoid otherwise slow convergence due to zigzag optimization trajectories.

(v) The bases of the found lattices provide the indexing solutions once the maxima of the score function have been found. A refinement step is then performed to minimize the mean Euclidean distance between the observed and predicted nodes using a gradient descent approach.

### 2.2. Relation between nodes and the indexing solution   

The Laue equations for a node **q**, the crystal lattice basis vectors **a**, **b**, **c**, and the Miller indices *h*, *k*, *l*, are defined as 
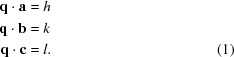
The nodes are the known observables, defined by the Bragg spots and the experimental parameters. The basis as well as the Miller indices need to be identified. Finding a solution to the above equation is equivalent to finding three linear independent solutions to the equation 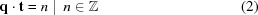
with a lattice basis vector **t**. Enforcing all *K* nodes found in the pattern to be on the reciprocal lattice by combining the nodes **q** into a matrix **Q** yields the following over-determined system of equations: 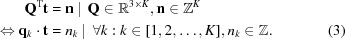



Each node 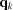 forms through equation (3) a series of equidistant parallel planes in the 3D space of **t** vectors distinguished and enumerated by the integers 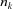. These are the planes of the real-space lattice of the crystal associated with the node. Any point on any of the planes is a solution to the equation formed by this node. The planes are orthogonal to 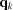 and their spacing is given by 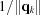. Different nodes form different sets of real-space planes; their intersections correspond to the real-space lattice, which are thus the points that solve equation (3). In an equivalent two-dimensional (2D) model, every node would form a series of equidistant parallel lines, as depicted in Fig. 2 ▸.

To solve equation (3), three linearly independent vectors must be found, where each vector points to one of the planes of each and every node (that is, to their intersections). Under real conditions, there are usually more than three nodes, making the problem over-determined. However, due to noise, the planes corresponding to these nodes will generally not all intersect at common points in real space (see the red node in Fig. 2 ▸), so an exact solution usually will not exist. The optimal solution **t** for equation (3) is therefore one that minimizes the average distance to one of the planes of each node. To find this solution we introduce a score function defined as a sum of proximity functions which themselves encapsulate the distance of the assumed solution from these geometrical planes.

### 2.3. Continuous proximity function for noise tolerance   

Every node defines a series of real-space parallel planes according to equation (3) and as sketched in Fig. 2 ▸ for the 2D case. Since the nodes are assumed to be noisy, the locations of the parallel planes also must be assumed to be noisy. This implies that the best estimate of the lattice basis vectors **t** (the optimal solution) might not exactly lie on the planes, but may instead lie close to the planes. Thus we define a 3D real-space proximity function c, that indicates how close a real-space vector is to a plane. This function is chosen to equal its maximum value at points on the planes, and is equal to its minimum value at points equidistant between two planes. A score function is then constructed for the entire arrangement of nodes as a normalized weighted sum of proximity functions given by 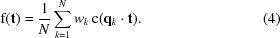
The weighting 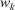 can depend on the intensities of the nodes, their norms, or other properties. The maxima of this score function are the feasible solutions of equation (3), corresponding to real-space lattice points. From these we obtain three linearly independent vectors to describe that lattice.

**Figure 3:**
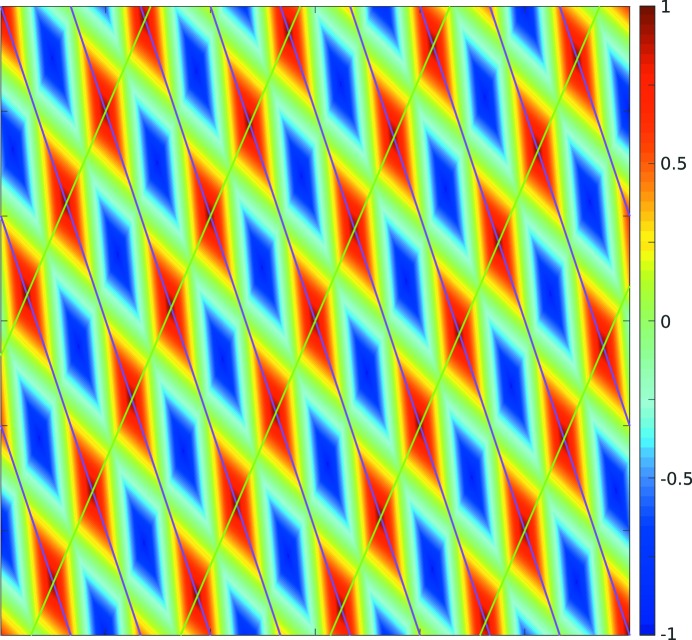
Score function for the lines from vectors *a* and *b* in Fig. 2 ▸. For each node, positions on the lines are assigned a proximity of 1 and positions in the middle between two lines are assigned the proximity −1. The rest of the proximity function for a single node is a linear interpolation of these values. The score function is formed by the normalized sum of the proximity functions of each node.

**Figure 4:**
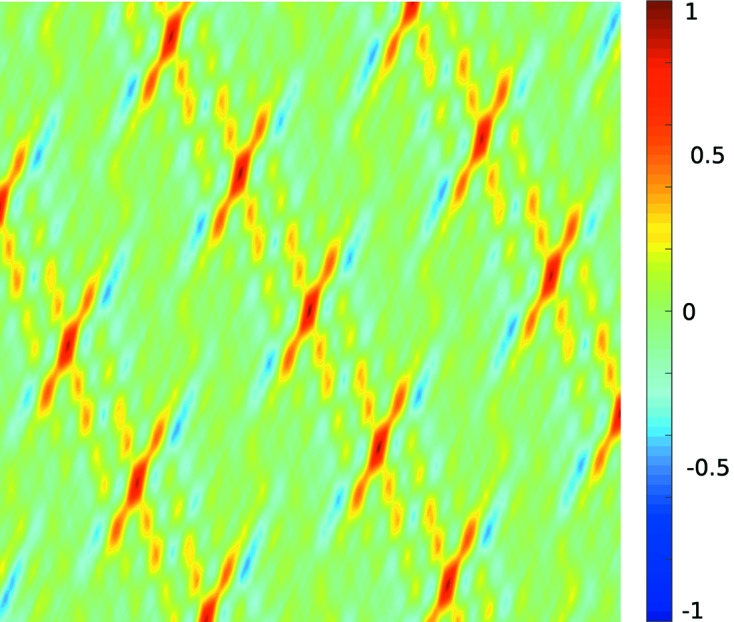
Score function for a set of 13 nodes that were generated by adding noise to the position of randomly chosen points on a lattice grid. For each node, positions on the lines are assigned a proximity of 1 and positions in the middle between two lines are assigned the proximity −1. The rest of the proximity function for a single node is a linear interpolation of these values. The score function is formed by the sum of the proximity functions of each node.

An example for the 2D case can be seen in Figs. 3 ▸ and 4 ▸. Fig. 3 ▸ shows the interpolation of the lines in Fig. 2 ▸ using proximity functions that vary linearly from their minimum to maximum values. Fig. 4 ▸ shows the score function of a sample arrangement of 13 nodes with this same choice of linear proximity functions.

As mentioned above, the proximity function indicates the distance from sets of parallel planes of equal spacing. While it is defined in 3D real space, it is a function only of distance along lines orthogonal to those planes. It is reasonable to define the proximity function to equal 1 on the planes, −1 midway between two planes, and to vary monotonically between these values. Combining these considerations, a proximity function of the following form is reasonable: 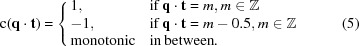
Many different functions are suitable for use as proximity functions. The execution time and thus the complexity of the function evaluation must be considered in its selection. The proximity function is periodic with a period of 1, so it can be defined in the interval [−0.5, 0.5] with 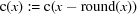. The following proximity functions are available in a tool-kit for further exploration and development of the program:

(*a*) 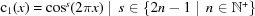.

(*b*) 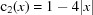.

(*c*) 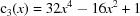.

(*d*) 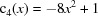.

(*e*) 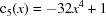.

(*f*) 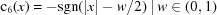.

(*g*) 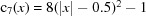.

(*h*) 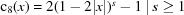.

**Figure 5:**
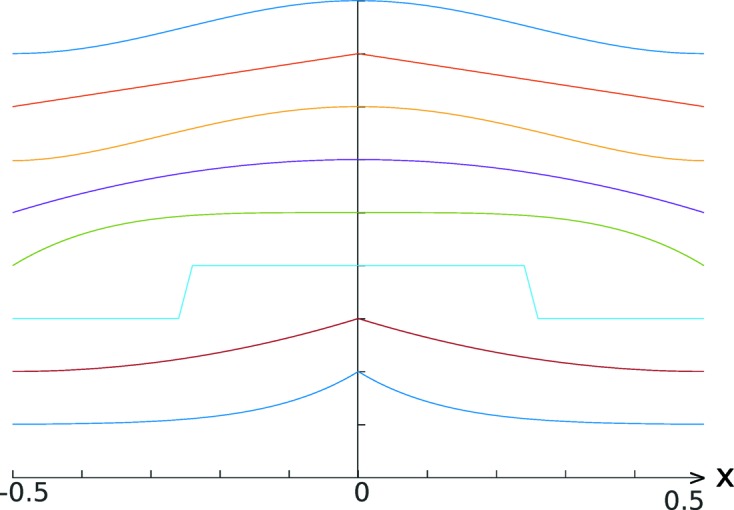
Plotted proximity functions 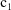 (top) to 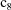 (bottom).

The proximity functions are visualized in Fig. 5 ▸. In the implemented heuristic we use only 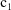 and 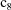.

It can be noted that using 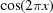 as a proximity function turns the score function [equation (4)] into the real part of the Fourier transform. Assuming that the geometry of the experiment is accurately known, the locations of the reflections in reciprocal space are centrosymmetric and so, if symmetrized, the Fourier transform of the arrangement of reflections would indeed be real. That is, the score function using 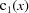 with 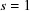 produces the Fourier transform of the given arrangement of reflections. Such a score function is used in the Fourier indexing methods, where lattice vectors are found by searching for maxima in the Fourier transform of a given arrangement of reflections. Our approach generalizes the use of a Fourier transform to that of an arbitrary proximity function. This extension provides a means to tune the proximity function to either achieve a greater noise tolerance (with a narrowly peaked function) or larger convergence radius for the search (with a broad function).

Not every Bragg spot found belongs to the same lattice. There may be false positives in the peak finding algorithm or peaks from different crystals in the same diffraction pattern. Such spurious peaks should ideally have as little impact as possible on the maxima of the score function. Their contribution can be removed by introducing a tolerance parameter 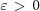. Nodes that generate planes that are too far away from the inspected vector are excluded from the computation of the score function. This distance of inclusion is given by ∊, so the smaller ∊, the more resistant the score function is to spurious peaks. The drawback of this method is that the score function can be discontinuous. The resulting score function is given by 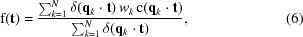
with 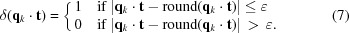
The solution to the indexing problem requires finding maxima of the score function. This is done by a local search in the 3D real space of the **t** vectors, which we aim to carry out efficiently to reduce computational time. The search must be started from a diverse number of starting points to ensure that more than one maximum is found. However, the search need only be conducted within a volume of the real space which can feasibly contain the real-space lattice vectors of the crystal. If the lattice parameters are not known in advance, then this volume can be restricted to a shell centred on the origin ranging in radius from the minimum to maximum possible lattice vector magnitudes, given reasonable assumptions. If the lattice parameters are known, then this search volume can be restricted considerably further, to spherical shells, each with a mean radius given by each of the real-space lattice parameters, as done by Gildea *et al.* (2014 ▸). The width of the shells is set to a tolerance that is dependent on the uncertainty of the lattice parameters.

The search is started simultaneously from a large number of evenly spaced points within the search volume that later migrate to the maxima of 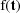 by a gradient descent approach. A typical number of starting points is 50 000. We achieve a set of starting points that are approximately uniformly separated and distributed throughout the volume of the spherical shell by first obtaining positions of points on a spherical surface that are approximately equally spaced from each other. This is done by minimizing a generalized electrostatic potential energy of a system of charged particles (Semechko, 2015 ▸). Since such computations can take a very long time, we use a set of pre-computed distributions of points on the unit sphere. This distribution is then scaled to several spherical surfaces that span the desired search shell. The radial increment of neighbouring surfaces is chosen to equal the average distance of neighbouring points on the sphere. To avoid systematic alignment of the points on each sphere, each point set is rotated about the origin in a random way.

While in theory it is sufficient to find the primary lattice vectors (*i.e.* the vectors of the reduced real-space lattice basis with Miller indices 100, 010 and 001), in the presence of spurious Bragg spots or multiple lattices we find that it is often beneficial to also search for the lattice vectors with Miller indices 110, 011 or 101. This is because spurious Bragg spots or spots from other lattices can significantly diminish some peaks in the score function. The use of additional lattice vectors adds redundancy and allows one to handle cases where the peaks in the score function belonging to the primary lattice basis vectors of a lattice are not detected. This procedure increases the execution time but improves the success rate of the algorithm, and is therefore provided as an option in our implementation of the algorithm.

Using even proximity functions 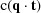, the score function 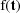 is centrosymmetric about the origin. We exploit this symmetry which allows the use of only half of the starting points.

### 2.4. Gradient descent extension   

We use an extended gradient descent method to let the starting points migrate to the maxima of 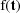.^1^ Empirical analysis shows that, for typical score functions 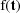 that we have employed, the gradient is often large at locations close to the maximum (see Fig. 4 ▸). The ordinary gradient descent method uses large step sizes for large gradients, which here is counterproductive. Instead, we generate a step size using a combination of the previous step length, the change in step direction, the value of 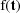, the number of well-fitted nodes, a parameter γ (as it is used in the ordinary gradient descent to regulate the relative step length), and clipping to a minimum and maximum step size. The parameters for the choice of the step size are empirically optimized and are not visible to the user.

As with the ordinary gradient descent algorithm, the problem occurs that convergence is often severely slowed down by zigzag migration trajectories (Wang, 2008 ▸). A common approach to overcome this problem is to use the conjugate gradient method (Hestenes & Stiefel, 1952 ▸). Given the known composition of our score function we instead use a different method. For every step we check whether the direction of the current step is nearly opposite that of the previous step. If this is the case, a zigzag path is probable and the current step direction is replaced by the sum of the unit-length vector pointing in the current direction and the unit-length vector pointing in the previous direction. This takes the search in a direction almost orthogonal to the previous ones, helping to overcome zigzag paths while being computationally very cheap.

### 2.5. Heuristic algorithm for locating maxima in the score function   

**Figure 6:**
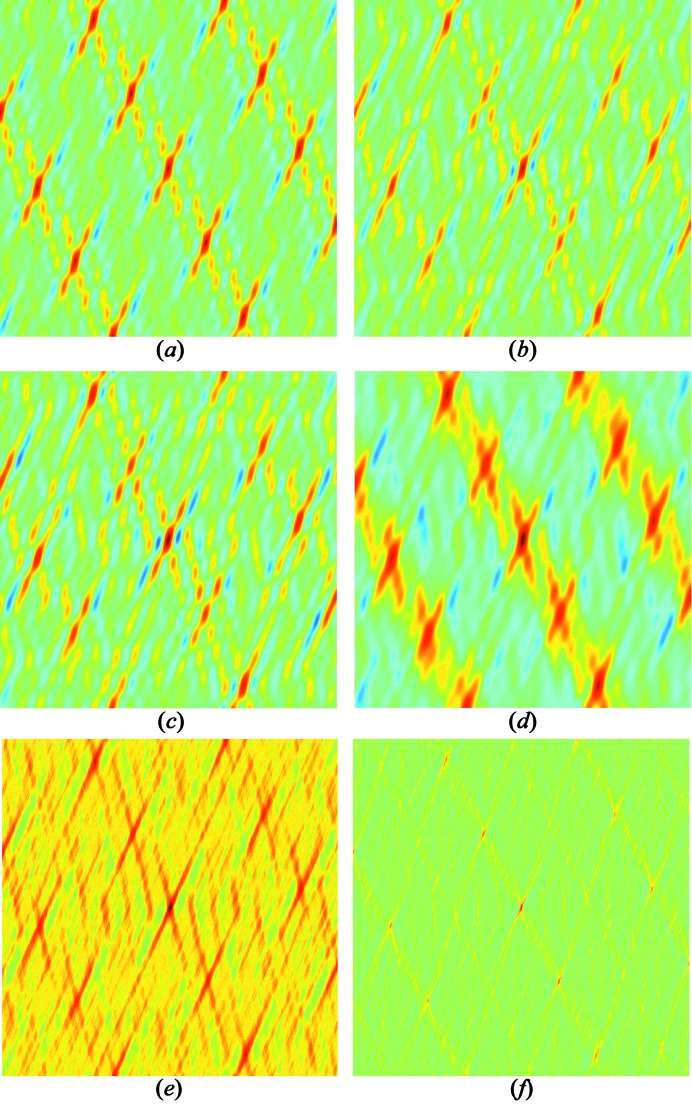
Score functions for a set of 13 simulated nodes that were generated by adding noise to the position of randomly chosen points on a lattice grid. For images (*b*)–(*f*) an additional seven spurious nodes were added, *i.e.* nodes not lying on the lattice. (*a*) Proximity function 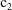, no spurious nodes. (*b*) Proximity function 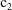. (*c*) Proximity function 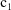. (*d*) Proximity function 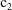 with inverse radial weighting. (*e*) Proximity function 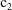 with score function from equation (6). (*f*) Proximity function 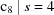 with score function from equation (6).

The goal of the heuristic is to find peaks in the score function, and hence probable lattice vectors, quickly and precisely. A large radius of convergence is required, but at the same time a very precise detection of the maxima is important. We therefore use a custom, empirically tuned algorithm with a multi-step design to home in on the maxima in stages. In this method, the earlier stages use smoother proximity functions, whereas in the later stages one with a sharper peak is used to achieve a more precise determination of the maxima:

(i) Gradient descent: proximity function 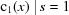, score function from equation (4), inverse radial weighting. [See Fig. 6 ▸(*d*) for visualization of the score function.]

The first stage is responsible for bringing the sampling positions close to the peak maximum without getting stuck in the local maxima. This is accomplished by using the Fourier transform proximity function 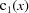 in conjunction with a weighting of the nodes proportional to 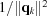. The radius-dependent weighting ensures a smooth score function by reducing weights of short-period proximity functions from high-resolution Bragg peaks, that otherwise would cause many local maxima. This stage is the most computationally expensive one, since it contains many gradient descent steps and operates on a large number of sampling points to ensure capturing the peak within the radius of convergence.

(ii) Gradient descent: proximity function 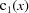, score function from equation (6). [See Fig. 6 ▸(*e*) for visualization of the score function.]

This and all subsequent stages bring the sampling points closer to their corresponding local maximum. These stages use the noise-tolerant and computationally expensive score function from equation (6), and unity weighting.

(iii) Gradient descent: proximity function 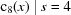, score function from equation (6), few steps. [See Fig. 6 ▸(*f*) for visualization of the score function.]

The third stage uses the very local and computationally expensive proximity function 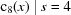. Using finer gradient descent steps, it is responsible to bring the sampling points close enough to the maxima to be able to identify even very sharp maxima by the score function evaluation at these sampling points.

(iv) Sparse peak finding on the sampling points.

Only the 50 sampling points with the highest score function evaluation in their respective local environment are kept. This drastically reduces the number of sampling points.

(v) Gradient descent: proximity function 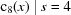, score function from equation (6), many steps. [See Fig. 6 ▸(*f*) for visualization of the score function.]

This last stage uses many fine gradient descent steps with the local and computationally expensive proximity function 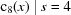, and with the score function of equation (6). This ensures that the sampling points migrate extremely close to the maxima, yet maintains an affordable computational effort due to the small number of sampling points used in this stage.

The numbers of steps for each stage can be chosen by a flag to the program.

A visualization of the employed score functions can be seen in Fig. 6 ▸, which shows score functions for a set of 13 simulated nodes that were generated by adding noise to the position of randomly chosen points on a lattice grid. For images (*b*)–(*f*) an additional seven spurious nodes were added, *i.e.* nodes not lying on the lattice. Despite the noise in the positions of the nodes, image (*a*) shows a high degree of periodicity. The additional seven spurious peaks significantly diminish some of the maxima in image (*b*). Image (*c*) shows slightly better contrast than image (*b*) at the expense of a more computationally expensive proximity function. Image (*d*) has a high radius of convergence for the gradient descent approach, but does not provide exact peak locations. Case (*e*) provides more accurate peak locations, but has a small convergence radius for the gradient descent approach. Case (*f*) uses a computationally expensive proximity function and suffers from a small convergence radius, but provides better noise-suppression capabilities and accurate peak locations.

### 2.6. Selection of lattice bases   

Once the maxima of the score function have been found, the bases of the found lattices can be formed. As a first step, all possible lattice bases are selected that each correctly predict at least five nodes. In theory, five nodes (given that they span the 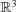) are more than what is minimally required to define a single lattice, but this increases the noise tolerance. The selection of candidate bases is computationally expensive, since there are 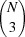 basis choices for *N* found peaks in the score function. To reduce computation time, as a first step all those vectors that predict less than five nodes are excluded. The next steps check for a reasonably high determinant of the basis and the number of correctly predicted nodes using two vectors and afterwards using three vectors. If the lattice parameters are known, the candidate lattices not fitting to these parameters are excluded as well. Finally, the basis vectors are sorted by the sum of each vector’s score function and the best 500 are kept.

Each kept candidate basis is reduced (to find the shortest vectors) using an efficient algorithm described by Semaev (2001 ▸). Afterwards, for each reduced basis the absolute defect (mean distance between the nodes and their positions predicted by the basis) and the relative defect (mean difference between the Miller indices of the nodes and the fractional Miller indices of the predicted nodes) are computed. From the 500 candidate lattices, 15 with the largest score function evaluation and 50 with the smallest relative defects are kept for the final stage.

In the final stage, the bases which best predict the nodes are selected. For this, the candidate bases are sorted in descending order by the number of nodes they correctly predict. Starting with the basis predicting the most nodes, it is considered as generating a true lattice if it either predicts at least five points that were not predicted by any other basis or if it has significantly smaller defects than a previously accepted basis. In the latter case, the newly found basis replaces the previous one. To avoid supercells in cases with unknown lattice parameters, bases with smaller determinants are preferred.

*XGANDALF* thus can detect several lattices in a diffraction pattern in one pass. This allows fast data processing despite the presence of several lattices in the pattern. If processing time is not of concern, employing the delete-and-retry technique (*i.e.* detect the strongest lattice in a pattern, delete the corresponding peaks and retry the indexing) can lead to better results. However, only this latter method is implemented in the interface to *CrystFEL* 0.8.0 (White *et al.*, 2012 ▸).

### 2.7. Refinement   

After the identification of the bases, a refinement step is performed. The lattice bases are refined to minimize the mean Euclidean distance between the nodes and the predicted nodes using a gradient descent approach. Only the nodes close to the predicted nodes are used for refinement to improve noise tolerance.

## 3. Evaluation of the algorithm   

### 3.1. Indexing rate   

Indexing solutions of measured diffraction patterns are often tested for correctness by comparing the locations of Bragg spots predicted by the lattice basis with those of the observed spots. If the pattern contains a large number of Bragg spots (say, 50) then this test usually yields a reliable estimate of correctness. If, on the other hand, the number of found spots is small then there can be several incorrect orientations of a crystal that predict the found spots, often giving a false indication of correctness. A reliable evaluation of the algorithm to index patterns as a function of the number of Bragg spots therefore requires ground truths, but ones which are as close as possible to real data. We generated our ground truths from a set of diffraction patterns which all had large numbers of Bragg spots, and as such were reliably indexed using *MOSFLM* (giving more than 50 correctly predicted peaks). The patterns were chosen from serial femtosecond crystallography data of crystals from serotonin receptor 5-HT_2B_ bound to ergotamine, publicly available from the CXIDB (Maia, 2012 ▸) entry 21 (Liu *et al.*, 2013 ▸). Patterns with fewer spots were created by simply deleting spots from these previously indexed patterns. This way we obtained realistic patterns with five to 50 spots, all with a known crystal orientation. We created two sets of patterns: one with the spots randomly distributed throughout the pattern, and the other with only low-resolution spots generated by removing Bragg spots from the original patterns at high scattering angles.

**Figure 7:**
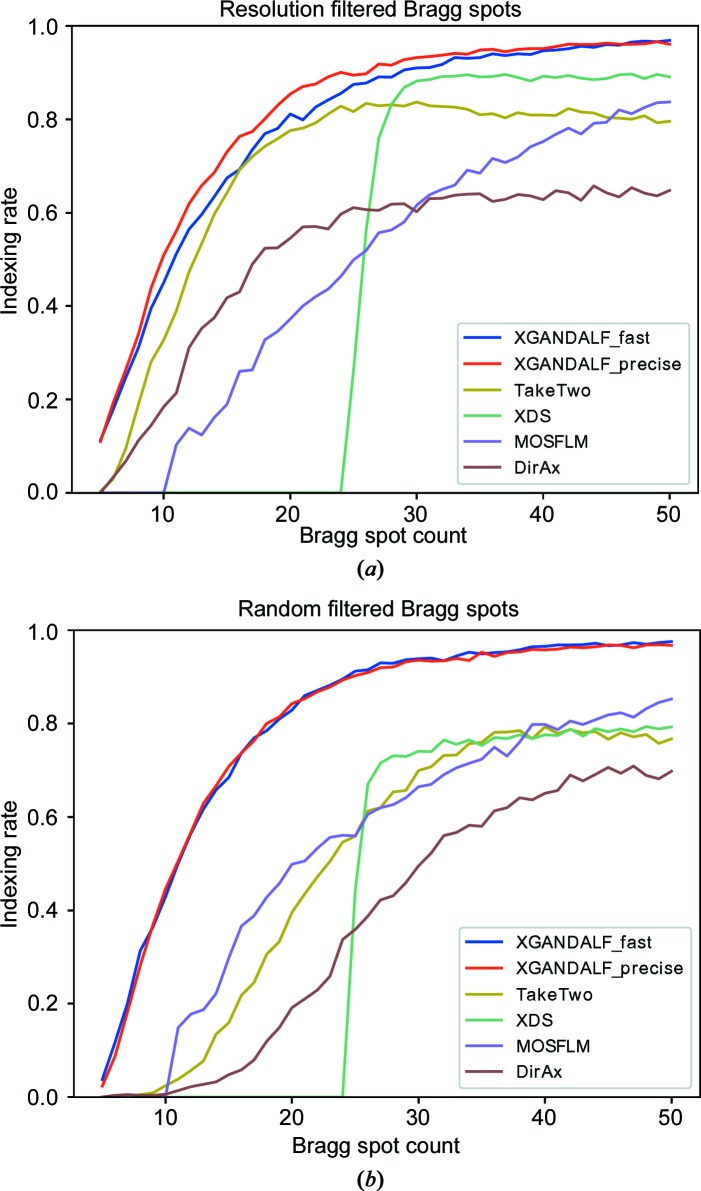
Comparison of the success rates of algorithms in indexing patterns as a function of the numbers of Bragg spots *N* in those patterns. The patterns were generated by selecting *N* Bragg spots from real diffraction patterns: (*a*) the *N* low-resolution Bragg spots were selected, (*b*) random *N* Bragg spots were selected. *XGANDALF* was used with ‘precise’ and with ‘fast’ settings. *XGANDALF* outperforms the other tested indexers over the whole range of Bragg spot counts in both (*a*) and (*b*).

To compare our indexing algorithm with others, the patterns from the two data sets were indexed using the *indexamajig* program from *CrystFEL* (White *et al.*, 2012 ▸). The use of *CrystFEL* allows a fair comparison of several (although not all) indexing algorithms with limited effort. The employed indexers are *MOSFLM* (Powell, 1999 ▸), *XDS* (Kabsch, 1993 ▸, 2010 ▸), *DirAx* (Duisenberg, 1992 ▸), *TakeTwo* (Ginn *et al.*, 2016 ▸), and two different modes of *XGANDALF*. One of these modes implemented many starting points and many gradient descent steps while the other mode used fewer starting points and fewer gradient descent steps. These are labelled, respectively, ‘XGANDALF_precise’ and ‘XGANDALF_fast’ in Fig. 7 ▸. In all cases the lattice parameters were specified to the indexing algorithm. No additional tuning of the indexing algorithms was performed. The indexing results were compared with the ground truths obtained from the original indexing of the patterns with *MOSFLM*. This comparison was accomplished by applying the Kabsch algorithm (Kabsch, 1976 ▸) to compute the angle needed to rotate one lattice basis onto another. Indexing solutions that required rotations of no more than 3° to bring them into coincidence with the ground-truth solution of *MOSFLM* (prior to removing spots) were counted as correct. For this test, all *CrystFEL* optimizations were turned off by using the options --no-retry --no-refine --no-check-cell. Only one indexing solution per pattern was accepted (using the option --no-multi). Although 3° is a significant deviation, this value is usually good enough for the subsequent refinement. For patterns with few peaks and a significant amount of noise, large deviations are anyway unavoidable. The results of the comparison are displayed in Fig. 7 ▸.

In a previous paper we remarked that *XDS* had a low success rate when indexing snapshot diffraction patterns (White, 2019 ▸), but due to parameter tuning and other improvements in the interface between *CrystFEL* and *XDS*, its success rate has been greatly improved with *CrystFEL* version 0.8.0 to be comparable with other algorithms, as indicated in Fig. 7 ▸.

The most practical test of indexing is the quality of the final merged data, as detailed in Section 3.3. Before that data can be merged, the full data set must be indexed. We used diffraction of beta-lactamase crystals from CXIDB ID 83 (Wiedorn *et al.*, 2018 ▸) for comparison. This data set consists of a total of 14 445 patterns identified as containing crystal diffraction, which were indexed by a variety of algorithms – the results are summarized in Tables 1 ▸ and 2 ▸. No additional tuning of the indexing algorithms was performed. Most patterns contained multiple hits, resulting in a total number of indexed crystals that for many indexers was higher than the number of patterns. Based on the experiment setup, the quality of the prediction and the quality of the merge results, it is most likely that these patterns really arose from multiple crystals. Although the unit-cell parameters were known, the indexing was processed in one case without providing that knowledge (only *MOSFLM*, *DirAx* and *XGANDALF* provided reasonably high indexing rates) and another with these parameters provided. Table 1 ▸ shows results for the case where the unit-cell parameters were not provided to the algorithms. In this case the indexers often report unit cells that differ from the known ones. For a fair comparison, the numbers of correctly identified unit cells are also listed. Table 2 ▸ presents the case using known unit-cell parameters.

As seen from Tables 1 ▸ and 2 ▸, *XGANDALF* outperforms all the other state-of-the-art indexers with and without prior cell information. Surprisingly, without prior cell information *XGANDALF* performs better in fast mode than in precise mode for this data set. It is likely that in the ‘precise’ mode more local maxima are found, making the choice for the basis selection algorithm more difficult.

### 3.2. Execution time   

For comparison of execution time, we took 1000 random patterns from the same data set of CXIDB ID 21 as described above in Section 3.1 and indexed them in the same fashion using the *CrystFEL* software suite on an Intel E5-2698 v4 CPU. Here, however, we did not remove spots from any of the patterns, nor did we select patterns only with a high number of Bragg peaks to create ground truths. The average number of Bragg peaks per pattern was 49. As before, the test was carried out for the two modes of *XGANDALF* – ‘XGANDALF_precise’ and ‘XGANDALF_fast’ – where parameters are chosen to either maximize the indexing success or maximize the indexing speed. Settings in between are also possible. The mean times to index the patterns are given in Table 3 ▸.

As can be seen in Table 3 ▸, again *XGANDALF* has the highest indexing rate among all tested indexers (in agreement with Fig. 7 ▸), while having an execution time of the same order of magnitude as the fastest-tested indexer. The high execution time for the *TakeTwo* algorithm reflects its mode of operation: if it does not find a solution, it will keep searching in the hope of eventually finding one, hence maximizing its indexing rate. Most patterns could be indexed by *TakeTwo* in a very short time, but several resulted in a long search. *CrystFEL* imposes a maximum running time on the indexing routines, and as a result the execution time shown for *TakeTwo* reflects this maximum time rather than the performance of the algorithm.

### 3.3. Final merged data quality   

**Figure 8:**
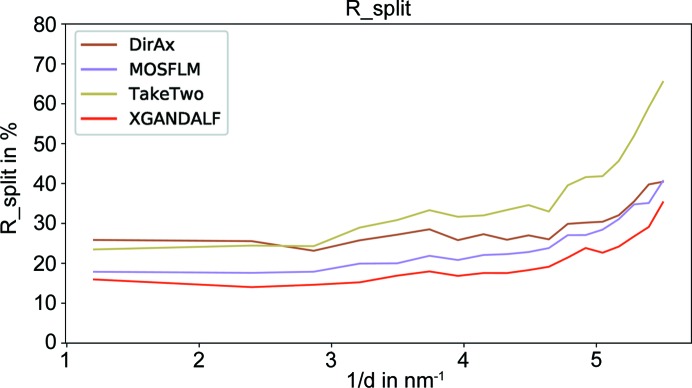
Comparison of the achieved 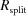 (White *et al.*, 2013 ▸) (lower is better) for *XGANDALF* and current state-of-the-art indexers. *XGANDALF* outperforms the other indexers in all significant resolution shells.

After indexing, the next stage in the processing pipeline is the merge of the measured Bragg spots of all patterns into a set of structure factors. Better indexing results should presumably lead to better statistics of the merged data, so the quality of the merge can be used as a measure of the quality of the indexing results. Here we merged the indexed data of CXIDB ID 83 that were summarized in Tables 1 ▸ and 2 ▸. Apart from the indexing algorithm selection, all parameters to *CrystFEL* were the same for all tests. For comparison of the merge results we used the figures of merit CC* (Karplus & Diederichs, 2012 ▸) and 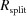 (White *et al.*, 2013 ▸). As shown in Figs. 8 ▸ and 9 ▸, *XGANDALF* significantly outperforms the other indexers in both of these figures of merit.

**Figure 10:**
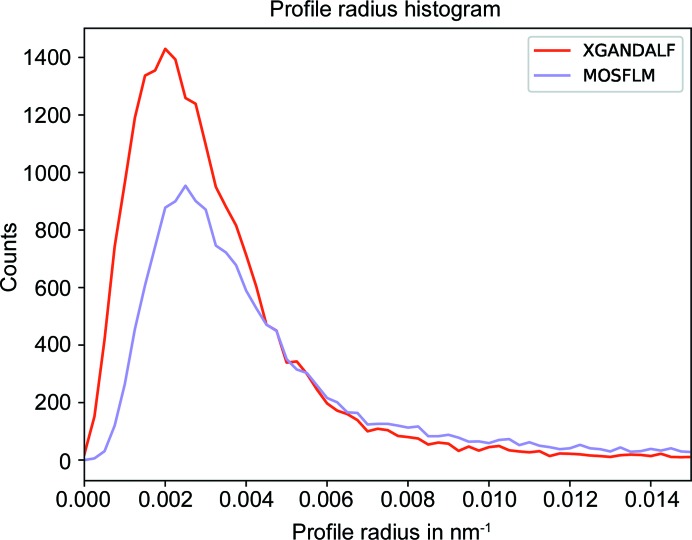
Comparison of the estimated profile radii of *MOSFLM* and *XGANDALF*. The estimated radii for patterns indexed by *XGANDALF* are generally smaller than the ones of *MOSFLM*, which means that the indexing solution is more precise.

For each indexed pattern *CrystFEL* (White *et al.*, 2012 ▸) estimates a profile radius of the Bragg spots. This is defined as the maximum distance of a reciprocal-lattice point to the Ewald sphere that still gives rise to a Bragg reflection, and can be considered as a property of the crystal, influenced by mosaicity for example. *CrystFEL* estimates this measure from the detected Bragg spots and the reciprocal-lattice points that predict them best. A similar metric, called the Ewald proximal volume, was used by Lyubimov *et al.* (2016 ▸) in their software *IOTA*. Errors in the indexing solution generally increase the estimated profile radius. A comparison of the profile radius estimation for *MOSFLM* and *XGANDALF* is depicted in Fig. 10 ▸. The estimated profile radii for patterns indexed by *XGANDALF* are generally smaller than the ones of *MOSFLM*, indicating that the indexing solution is more precise.

## 4. Availability   

*XGANDALF* is implemented as an open-source C++ library, which can be used directly from applications written in C or C++, or from a Python program using a Cython interface. *XGANDALF* has been implemented in *CrystFEL* (White *et al.*, 2012 ▸) and is available from version 0.8.0 onwards. The *XGANDALF* implementation provides the tools for programmers to adjust the heuristic by defining their own high-level heuristic stages based upon optimized low-level implementations. The library is distributed under the LGPLv3 licence, and the source code can be downloaded from https://stash.desy.de/users/gevorkov/repos/xgandalf/browse.

## 5. Conclusion   

A new indexing algorithm, *XGANDALF*, has been presented which was designed specifically for indexing still diffraction patterns for snapshot serial crystallography experiments. As such, it outperforms the current state-of-the-art indexers that, although commonly used in serial crystallography, were mostly created for the indexing and analysis of rotation crystal data. Compared with those programs, *XGANDALF* gives higher indexing rates and higher indexing precision, and can be used both with and without prior unit-cell parameters. The execution time of the implementation is of the same order of magnitude as currently used indexing algorithms and, with mean indexing times of about 20 ms, is fast enough to allow real-time feedback in experiments. Compared with the available indexers, the algorithm successfully indexes more patterns in test serial crystallography data sets and is more robust to multiple lattices in a single image. The program has already been used in serial crystallography experiments by several other groups with very positive results. We therefore anticipate that *XGANDALF* will be a valuable addition to the collection of software tools for serial crystallography.

## Figures and Tables

**Figure 9:**
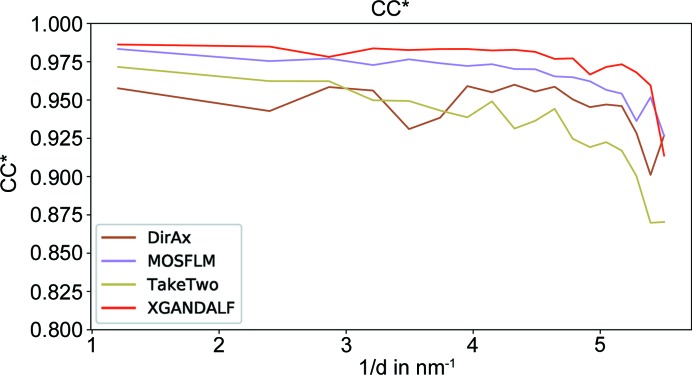
Comparison of the achieved CC* (Karplus & Diederichs, 2012 ▸) (higher is better) for *XGANDALF* and current state-of-the-art indexers. *XGANDALF* outperforms the other indexers in all significant resolution shells.

**Table 1:** Numbers of crystals of CXIDB ID 83 indexed without prior unit-cell knowledge

Indexer (no prior cell information)	Total indexed	Indexed with correct unit cell
*DirAx*	28 832	3553
*MOSFLM*	18 346	11 742
*XGANDALF* fast mode	26 040	14 631
*XGANDALF* precise mode	24 748	10 899

**Table 2:** Numbers of crystals of CXIDB ID 83 indexed with prior unit-cell knowledge

Indexer	Indexed
*XDS*	13 922
*MOSFLM*	16 120
*MOSFLM DirAx XDS*	17 433
*TakeTwo*	18 808
*XGANDALF* ‘fast’ mode	19 914
*XGANDALF* ‘precise’ mode	21 171

**Table 3:** Comparison of mean execution times (per pattern) and indexing results for a data set consisting of 1000 patterns

Indexer name	Indexed patterns	Mean execution time (ms)
*MOSFLM*	452	17
*XDS*	400	22
*DirAx*	394	12
*TakeTwo*	545	662
*XGANDALF* fast mode	724	19
*XGANDALF* precise mode	725	106

## Footnotes

1 Strictly, we minimize the negative of the score function by gradient *descent*.
